# Happiness and mental health of older adults: multiple mediation analysis

**DOI:** 10.3389/fpsyg.2023.1108678

**Published:** 2023-04-26

**Authors:** Yujin Sun

**Affiliations:** School of Management, Suzhou University, Suzhou, China

**Keywords:** happiness, health, mental health, mediation analysis, healthy aging

## Abstract

**Introduction:**

This study aims to explore the influence mechanism of older adult mental health. As the aging population grows, the mental health of older adults becomes a significant public health and social issue, with happiness serving as a key dimension of mental health.

**Methods:**

This study utilizes public data from CGSS to investigate the relationship between happiness and mental health, with Process V4.1 used for mediating effects analysis.

**Results:**

The findings suggest a positive predictive effect of happiness on mental health, with three mediating paths identified: independent mediating effect paths of income satisfaction and health, as well as a multiple mediating effects path through income satisfaction and health.

**Discussion:**

The study suggests that improving the multi-subject mental health support service system for older adults and creating public values for mental health risk coping mechanisms. This helps to understand the complex relationship between aging on individual and social levels. These results provide empirical support for healthy aging among older adults and future policymaking.

## 1. Introduction

From PRB’s 2020 world population data, the proportion of individuals aged 65 and older is 9% worldwide, 19% in developed countries, 16% in the United States, 22% in Germany, 14% in East Asia, 29% in Japan, 16% Republic of Korea, and 13.5% in China (PRB’s 2020 World Population Data Sheet, 2022). The United Nations predicts that by 2050, over 16% of the world’s population will be over 65 years of age, and over 25% in Europe and North America, Japan 37.5%, China will reach 40.6% (World Social Report, 2023). With the increasing prevalence of aging, older adults face multiple challenges including caregiving, intergenerational separation, digital divide, frailty, and chronic diseases (Fried et al., 2001; James et al., 2018). The issue of aging is of great academic and research value, with mental health problems being of particular concern as the mental health of older adults is declining year by year (Batsis et al., 2021) and is significantly related to the mortality rate of older adults (Lee, 2000).

Happiness and mental health are closely related concepts (Bray and Gunnell, 2006), and the Dual-factor model of mental health suggests that happiness is an important indicator of mental health (Greenspoon and Saklofske, 2001). Happiness is the foundation of mental health, as mental health is a by-product derived from happiness (Yu, 2022). Functionally, happiness is a prerequisite for mental health, and its impact on mental health is mediated through intermediaries (Zeidner et al., 2016). Happiness is closely related to health, and older adults with greater happiness are more likely to disseminate health information (Greiner et al., 2004). The positive relationship between happiness and health has been well-documented in empirical studies conducted in Italy and Norway (Straume and Vittersø, 2015). This relationship suggests that happiness serves as an important predictor of health levels, implying that there is a pathway through which happiness can impact individual health (Sabatini, 2014). This study aims to examine the relationship between happiness and the mental health of older adults, as well as its influencing factors and mechanisms, to enhance the understanding of aging.

Research suggests that people with higher levels of happiness also report higher levels of income satisfaction (Graham et al., 2004) and that happiness affects employment probability (Krause, 2013). Moreover, older adults with higher levels of happiness have a greater probability of good health, and instrumental variables are used to address endogeneity problems in studies of this relationship (Zhu and Yang, 2017). The decline in physiological function among older adults leads to a reduction in their ability to obtain income, making them susceptible to falling into poverty (Ku and Kim, 2020). Income is crucial in enabling older adults to access medical services, and higher income levels are associated with better health outcomes (Pulok and Hajizadeh, 2022). Economic poverty is closely linked to the health of older adults, with poverty increasing the risk of health problems and economic disparities exacerbating health inequality (Liu et al., 2022). Income is a significant indicator of social and economic status, and pension can have a direct impact on the mental health of older adults by providing them with a stable source of income (Case and Wilson, 2000). Higher income levels and better economic circumstances can reduce the level of depression among older adults (Abe et al., 2012), while poverty has adverse effects on their mental health (Ervin et al., 2021). The association between income inequality, income satisfaction, and mental health among older adults is well-established (Wang et al., 2022), and there are socioeconomic gradients of mental health (Yahirun et al., 2020; Srivastava et al., 2021).

Healthy life expectancy was proposed by Sanders (1964), emphasizes the importance of maintaining good health while extending life (Payne and Xu, 2022). The decline in physical health among older adults is a persistent trend, resulting in physical weakness, disease, and functional disability that can cause mental health problems and increase the risk of suicide (Fässberg et al., 2016). The older adults with chronic diseases or poor health are more likely to be lonely, and the older adults with deteriorating health have a greater risk of mental health problems (Emerson and Jayawardhana, 2016). Disability is significantly related to mental health (Verhaak et al., 2014), with an increase in depression index observed among older adults with disabilities (Yan et al., 2023). A study found that the incidence of depressive symptoms of the disabled older adults aged 70 in the UK is 2.8 times that of healthy people and that of Greece is 2.2 times (Holmquist et al., 2017). Life course is composed of events and experiences in the process of human development. The internal driving force and development factors affecting the life course of the older adults are worth exploring (Mayer, 2009). The older adults are faced with double vulnerabilities of economy and health, easy to fall into the trap of “poverty and illness” (Xia and Liu, 2018). Research on income and health (Zhang et al., 2023), as well as their effects on mental health (Zhu and Chou, 2022), is essential in the context of population aging. Additionally, urban and rural factors, gender factors, and education level can affect the mental health of older adults (Hu and Huang, 2019).

Elder’s life course theory posits that individuals construct their life course through their choices and actions, taking advantage of opportunities (Elder, 2018). Life course theory pays attention to the relationship between individuals and socio-economic situations. Happiness, as a personal feeling, is an essential component of the daily life of older adults, and mental health is a crucial public health and social issue. Understanding the intricate relationship between happiness and mental health, and the potential mediating roles of income and health, is important in enhancing academic cognition of mental health among older adults, has practical value (Steptoe et al., 2015). It is helpful to understand the complex relationship between aging at the individual and social level, and to provide empirical support for the healthy aging of the older adults and future policymaking.

## 2. Materials and methods

### 2.1. Data sources

The study utilizes data from the Chinese General Social Survey (CGSS) conducted in 2017, a comprehensive survey that covers a wide range of aspects including health, population, social economy, and psychology. The CGSS is well-suited for large-scale national research and allows for the measurement of health and mental health, as well as exploring the internal link mechanism at different levels. The research focuses on older adults aged 60 years and above. After data collation and screening, there are 4,372 valid samples retained. The sample comprises 2,121 males (48.5%) and 2,251 females (51.5%), with 2,594 urban older adults (59.3%) and 1,778 rural older adults (40.7%). The Cronbach’s α is 0.738, and the KMO is 0.726, which indicates good reliability and validity of the data.

### 2.2. Measurements

The outcome variable mental health was measured by the question “In the past 4 weeks, how depressed or frustrated did you feel?,” always assigned to 1, often assigned to 2, sometimes assigned to 3, rarely assigned to 4, never assigned to 5, higher scores on this item are indicative of better mental health (Ran and Hu, 2022). The predictor variable happiness was measured by the question “In general, do you feel that you are happy in your life?,” from very unhappy to very happy assigned 1–5, with the higher score the higher level of happiness (Zhu and Yang, 2017). Previous studies have included health, income, and population aging in the analytical framework to construct models (Liu and Sun, 2022), and this study chose income satisfaction and health as mediating variables. Income satisfaction is assessed by asking about their satisfaction with family income, with higher scores indicating greater satisfaction (Wang et al., 2022). Health is measured by a self-assessment of physical health (Bryant et al., 2000), which is measured by “How do you feel about your current physical health?” and is assigned a score of 1–5 from very unhealthy to very healthy, with higher scores indicating better health. Based on previous literature, gender, education level, and urban-rural factors were included as control variables (Lu and Wang, 2022).

### 2.3. Statistical methodology

The present study employs data from CGSS to investigate the relationship between happiness and mental health. Descriptive statistics and correlation analysis were conducted using SPSS26, and mediating effect analysis was carried out using PROCESS V4.1 (Hayes, 2017). To analyze the multiple mediations, PROCESS model 6 was utilized with the non-parametric estimation bootstrap method to test the significance of mediations. Repeated sampling of 5,000 was used to calculate the 95% confidence interval.

## 3. Results

### 3.1. Comparison of variable differences

The results presented in Table 1 indicate gender-related disparities in health and mental health outcomes. Moreover, there are noteworthy variations in happiness, income satisfaction, health, and mental health between urban and rural areas. By dividing the samples into high and low happiness groups, differences were observed in income satisfaction, health, and mental health, with effect sizes measured using Cohen’s d revealing significant distinctions.

**TABLE 1:** Difference comparison results of each variable.

	Male (M ± SD)	Female (M ± SD)	*t*	Cohen’s d
1 Health	3.08 ± 1.071	2.87 ± 1.072	6.580**	0.196
2 Mental health	3.83 ± 0.997	3.64 ± 1.052	5.867**	0.185
	**Urban (M ± SD)**	**Rural (M ± SD)**		
1 Happiness	3.97 ± 0.806	3.83 ± 0.914	5.027**	0.162
2 Income satisfaction	4.02 ± 1.229	3.54 ± 1.363	6.800**	0.37
3 Health	3.13 ± 1.034	2.75 ± 1.098	11.515**	0.356
3 Mental health	3.91 ± 1.015	3.47 ± 0.996	14.082**	0.438
	**Low happiness (M ± SD)**	**High happiness (M ± SD)**		
1 Income satisfaction	2.84 ± 1.197	4.10 ± 1.199	16.470**	−1.052
2 Health	2.54 ± 1.026	3.09 ± 1.060	13.954**	−0.527
3 Mental health	3.16 ± 1.042	3.88 ± 0.973	18.802**	−0.714

**P < 0.01.

### 3.2. Correlation matrix between variables

The analysis results presented in Table 2 reveal a statistically significant positive correlation among the variables of happiness, income satisfaction, health, and mental health. This correlation serves as a basis for testing the mediating effect.

**TABLE 2:** Correlation matrix of happiness, income satisfaction, health, and mental health.

	M	SD	1	2	3	4
1 Happiness	3.91	0.854	–			
2 Income satisfaction	3.82	1.306	0.456**	–		
3 Health	2.97	1.077	0.247**	0.242**	–	
4 Mental health	3.73	1.03	0.343**	0.309**	0.435**	–

**P < 0.01.

### 3.3. Mediating effect test

This study aimed to examine the relationship between happiness and mental health, mediated by income satisfaction and health, and controlled for gender, education level, and urban and rural factors. Table 3 shows that three model can be constructed. First, happiness is the result variable, income satisfaction is the prediction variable (β = 0.663, *P* < 0.001), *R*^2^ = 0.231; Second, happiness (β = 0.249, *P* < 0.001), and income satisfaction (β = 0.088, *P* < 0.001) positively predicted health, *R*^2^ = 0.145; Third, taking mental health as the outcome variable, happiness (β = 0.243, *P* < 0.001), income satisfaction (β = 0.084, *P* < 0.001), and health (β = 0.321, *P* < 0.001) had significant positive predictive effect, *R*^2^ = 0.313.

**TABLE 3:** Regression analysis among variables.

Model		Overall model fit			Significant	
**Outcome variable**	**Predictor variable**	**R**	***R* ^2^**	**F**	**β**	***t***
Income satisfaction	Happiness	0.48	0.231	105.451	0.663	18.673**
	Gender				0.065	1.037
	Level of education				0.028	2.051*
	Urban and rural				0.311	−4.596**
Health	Happiness	0.38	0.145	47.6	0.249	7.434**
	Income satisfaction				0.088	3.908**
	Gender				0.120	−2.270*
	Level of education				0.055	4.743**
	Urban and rural				0.267	−4.642**
Mental health	Happiness	0.559	0.313	106.556	0.243	8.079**
	Income satisfaction				0.084	4.202**
	Health				0.321	13.671**
	Gender				0.142	−3.035**
	Level of education				0.024	2.287*
	Urban and rural				0.286	−5.584**

*P < 0.05; **P < 0.01.

The results of the non-parametric Bootstrap test in Table 4 indicated that the mediating effect was significant, with a total indirect effect of 0.154. Three mediating chains were identified: happiness ^→^ income satisfaction ^→^ mental health, happiness ^→^ health ^→^ mental health, and happiness ^→^ income satisfaction ^→^ health ^→^ mental health. The relative mediating effects of these paths were 13.85, 20.15, and 4.79%, respectively. Therefore, income and health play a significant chain mediating role between happiness and mental health.

**TABLE 4:** Mediating effects of income satisfaction and health between happiness and mental health.

	Effect	BootSE	BootLLCI	BootULCI	Relative effect
Direct effect	0.243	0.030	0.184	0.302	61.21%
Happiness → income satisfaction → mental health	0.055	0.015	0.026	0.086	13.85%
Happiness → health → mental health	0.080	0.013	0.056	0.107	20.15%
Happiness → income satisfaction → health → mental health	0.019	0.005	0.008	0.030	4.79%
Total indirect effects	0.154	0.020	0.115	0.194	38.79%
Total effect	0.397	0.028	0.341	0.452	

**FIGURE 1:**
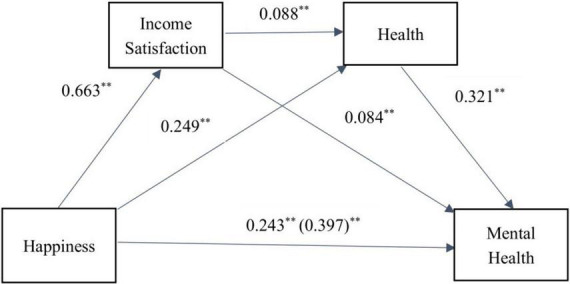
Chain mediation effect path diagram. **P < 0.01.

The findings of this study provide important implications for improving mental health by enhancing happiness, income satisfaction, and health. A visual representation of the mediating model is presented in Figure 1.

### 3.4. Robustness test

Given that the CGSS2017 dataset used in this study is cross-sectional, robustness tests were performed to mitigate the risk of endogeneity.

#### 3.4.1. Change the sample size

Selective sampling techniques were employed, whereby the samples of unhealthy older adults were removed and the chain mediation effect of the remaining samples was examined. The results of this test showed that the research conclusion remained valid. Further robustness tests were conducted by eliminating samples with poor physical and mental health, while keeping the dependent, independent, mediator, and control variables constant. Subsequent mediation tests yielded the same three equations with positive prediction effects, and the mediation effects remained significant, albeit with slightly reduced influence coefficients. The Bootstrap test confirmed that the research results were not subject to significant endogeneity risks.

#### 3.4.2. Variable substitution

The CGSS data contains queries about family economic status and health impact, which were used as replacements for income satisfaction and health, respectively. After testing, the β and effect values exhibited slight changes, but the three mediation effects remained significant and the research results were consistent. This further strengthens the robustness of the research conclusion.

## 4. Discussion

### 4.1. Comparison of variables

The present study compared various variables and found consistent results with previous research. Results indicate that male older adults have better mental health than females (Kiely et al., 2019), older adults in high-income countries have better mental health than those in middle-income countries, and rich older adults have significantly higher mental health status than poor older adults (Jiang and Gao, 2021). There are also noticeable differences in mental health between urban and rural areas, with urban older adults having significantly higher mental health levels than rural older adults (Sun and Lyu, 2020). This may be due to the lack of spiritual care in rural areas where older adults are left alone. The study also found income differences between urban and rural areas and significant differences in income satisfaction between urban and rural older adults (Mitra et al., 2020). The study suggests that the health level of urban older adults is better than that of rural older adults due to the imbalance of medical resources between urban and rural areas (Ran and Hu, 2022). The study concludes that health inequality is a form of social injustice that requires attention (He and Shen, 2021), and public policies should be implemented to bridge the gap between urban and rural areas and promote the health equality of older adults. Additionally, the study found differences in happiness between urban and rural residents and that happiness is divided into high and low groups (Wang et al., 2014), with significant differences in income satisfaction (Zhang, 2021). The results also suggest that there is no Easterlin paradox among older adults.

### 4.2. Correlation analysis among happiness, income satisfaction, health, and mental health

The findings indicate a substantial and positive association between happiness and income satisfaction. Data analysis of the World Poll reveals that happiness is positively linked with per capita GDP, and that individuals in affluent nations tend to report higher levels of happiness (Deaton, 2007). There is a correlation between happiness and health, as happiness is known to have a beneficial effect on health (MacKerron, 2012). Income is also known to impact health status (Kahn and Fazio, 2005), with health being a significant factor in determining income as a component of human capital (Cheng et al., 2014). Moreover, the health of older adults is closely linked to their economic and social status (Rehnberg, 2020). Income is significantly associated with mental health (Gero et al., 2017), and an increase in economic status can enhance the mental health of older adults (Lei et al., 2014). Poverty is a significant predictor of cognitive impairment and depression among older adults (Reddy et al., 2012), and it can result in stress and negative emotions, leading to a detrimental impact on mental health (Haushofer and Fehr, 2014). The correlation findings of each variable are in agreement with prior research.

### 4.3. Mediating effect between income satisfaction and health

The study reveals a significant mediating effect path between happiness, income satisfaction, and mental health, where income satisfaction serves as a mediator. This finding is consistent with prior research showing that happiness positively affects income (Graham et al., 2004) and work efficiency (Peterson et al., 2011), and that income satisfaction is associated with better mental health (Park and Seo, 2021). Moreover, the study finds a significant path among happiness, health, and mental health, with health acting as a mediator, in line with the bio-psychological-social model of aging (Cosco et al., 2013). People with higher levels of happiness tend to have better health habits, live longer (Frey, 2011), and experience fewer health risks (Diener and Chan, 2011). The study also identifies a chain mediating effect path between happiness, income satisfaction, health, and mental health, which can be explained by SOC. The older adults tend to optimize their resources in important areas such as happiness, income satisfaction, and health, thereby improving their mental health and reducing the risk of mental health problems (Baltes and Baltes, 1990). Research shows that disabled older adults in rural areas facing multiple difficulties are particularly vulnerable to health inequality, which can lead to mental health crises (Hu and Wang, 2019; Li and Bai, 2022). The research thus provides important insights into the complex interplay among happiness, income, health, and mental health in the aging process.

### 4.4. Implications

#### 4.4.1. Work together to build a healthy aging society

Enhancing the mental health support service system for the older adults and raising their mental health level requires collaboration among the state, community, and family (Yuan and Jin, 2021). Creating a public value for coping with mental health risks among the older adults necessitates a consensus on the formation of rights, obligations, and norms, as per Bozeman’s notion of Public Values (Bozeman, 2007). Improving the mental health level of the older adults can reduce the risk of death (Lee, 2000) and is their right, as well as the responsibility of the state and obligation of the family. Focusing on the older adults is a principle that social operation should follow, and a consensus-oriented public value is the way to handle the mental health risk of the older adults (Andersen et al., 2013).

#### 4.4.2. Caring for the older adults

Social-emotional selectivity theory suggests that in old age, they require more intimate relationships, care from families, and interaction with social peers to meet their spiritual needs (Charles and Carstensen, 2007). It is important to provide emotional support for older adults, and the level of public cultural services should be improved to provide more humanistic care and achieve high-quality aging. Focus on sustainable livelihoods for the older adults (Zhang et al., 2018). Protecting financially challenged older adult individuals (Quinn and Cahill, 2016), improving their social capital and employability, encouraging their active participation in labor, enhancing their income satisfaction and sense of acquisition, and actively responding to aging (Pack et al., 2019). Promoting healthy aging involves using new media to disseminate health information, promoting correct understanding of health problems and aging processes among the older adults, encouraging them to engage in preventive health behaviors, appropriate physical exercise activities, consume enough healthy nutrition, manage their lifecycle health, and establish healthy lifestyles (Levy and Myers, 2004). Public spaces should be provided to meet the daily activities of the older adults, enabling them to have a happy old age and improve their healthy life expectancy.

#### 4.4.3. Improving the security system for older adults

Improving the social security system, especially the old age security system, promoting the equalization of public medical services, and establishing a social support service system for older adults are important steps toward a healthy aging society (Teater and Chonody, 2020). As we improve the social security of older adults, exploring the positive and growth potential of aging is necessary (Scheibe and Carstensen, 2010). Attention to mental health support for older adults is critical, and enhancing their mental health risk coping mechanism can enable them to relieve stress in the face of difficulties, provide spiritual comfort and psychological intervention services, and improve their mental health level. Promoting the psychological resilience of older adults to cope with risks, adapt well, and cope successfully with aging can encourage successful aging with healthy and positive aging (Pruchno and Carr, 2017).

### 4.5. Limitations

The study has passed the robustness test and reduced the risk of endogeneity. However, due to the cross-sectional nature of the CGSS 2017 data, it is impossible to determine the order of appearance of happiness and mental health. Therefore, variables may have a reverse causal relationship. In the future, longitudinal studies can be conducted to verify the causal relationship between happiness and mental health. The influencing mechanism of mental health among older adults is complex and requires further exploration of other influencing mechanisms.

## 5. Conclusion

This study utilized CGSS public data to investigate the mechanism that influences mental health in older adults. The results indicate that happiness, income satisfaction, health, and mental health are significantly and positively correlated. Happiness has a positive predictive effect on mental health. The impact of happiness on mental health is mediated through three paths: an independent mediating effect path of income satisfaction, an independent mediating effect path of health, and a multiple mediating effect path through income satisfaction and health. These findings suggest that happiness, income satisfaction, and health in old age are better indicators of the value goal of mental health. This study mainly focuses on the relationship between happiness and mental health from the perspective of healthy aging, which has strong practical significance. The impact mechanism discovered by this study can provide a basis for policymaking, caring for older adults, focusing on sustainable livelihoods for older adults, improving social security systems, enhancing older adults’ mental health support service systems, and helping to actively respond to aging challenges.

## Data availability statement

Publicly available datasets were analyzed in this study. This data can be found here: http://cgss.ruc.edu.cn/index.htm.

## Author contributions

YS designed the study, performed the statistical analysis, wrote the first draft, polished the manuscript, and approved the submitted version.
